# Supplementary material for: C7-Prenylation of Tryptophan-Containing Cyclic Dipeptides by 7-Dimethylallyl Tryptophan Synthase Significantly Increases the Anticancer and Antimicrobial Activities
Source: Molecules. 2020 Aug 12;25(16):3676. doi: 10.3390/molecules25163676 (PMC7463755; doi:10.3390/molecules25163676)
Supplement: Supplementary file 1 [file molecules-25-03676-s001.pdf]

Supplementary Material

A significant increase of antiproliferative, antibacterial and antifungal activity of C7-prenylation in tryptophan-containing cyclic dipeptides by 7-dimethylallyl tryptophan synthase

Rui Liu [1,2], Hongchi Zhang [1,2*], Weiqiang Wu [2], Hui Li [1,2], Zhipeng An [2], Feng Zhou [2]

[1] College of Life Science, Shanxi Datong University, Datong Shanxi 037009, China;
[2] Applied Biotechnology Institute, Shanxi Datong University, Datong Shanxi 037009, China
* Correspondence: zhanghclw@163.com

Figure S1. Dependency of the product formation on DMAPP, 1a-7a concentrations. Michaelis-Menten equation, Lineweaver-Burk, Hanes-Woolf, and Eadie-Hofstee plots of DMAPP, 1a-7a

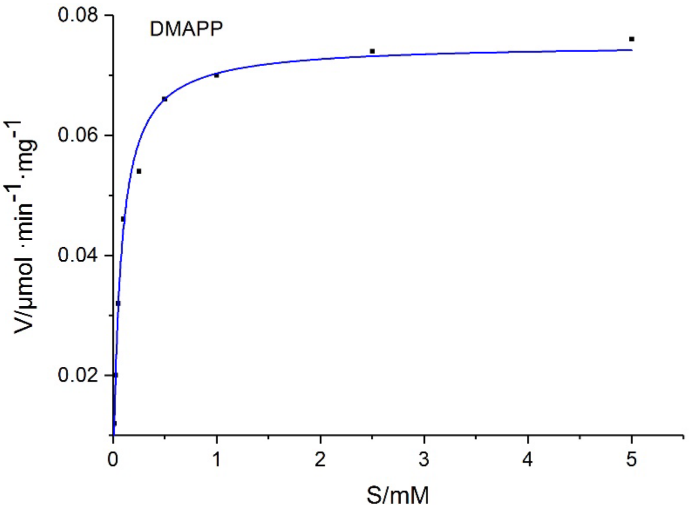

Michaelis-Menten equation

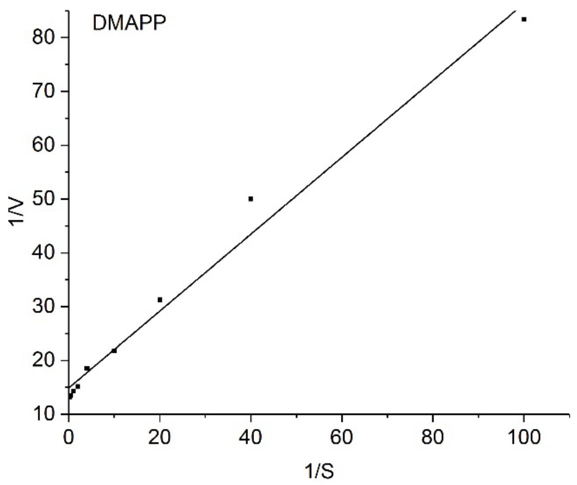

Lineweaver-Burk

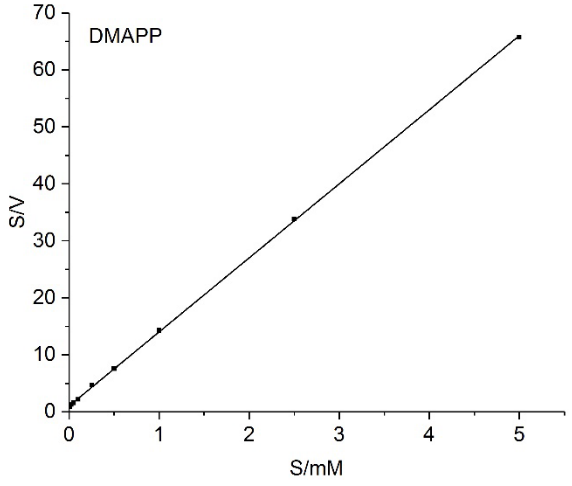

Hanes-Woolf

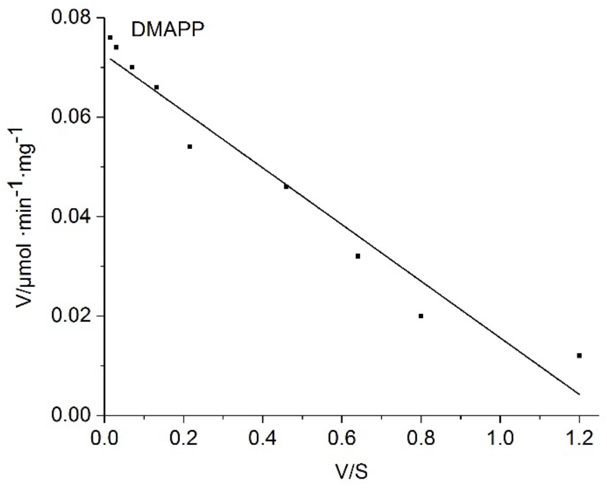

Eadie-Hofstee

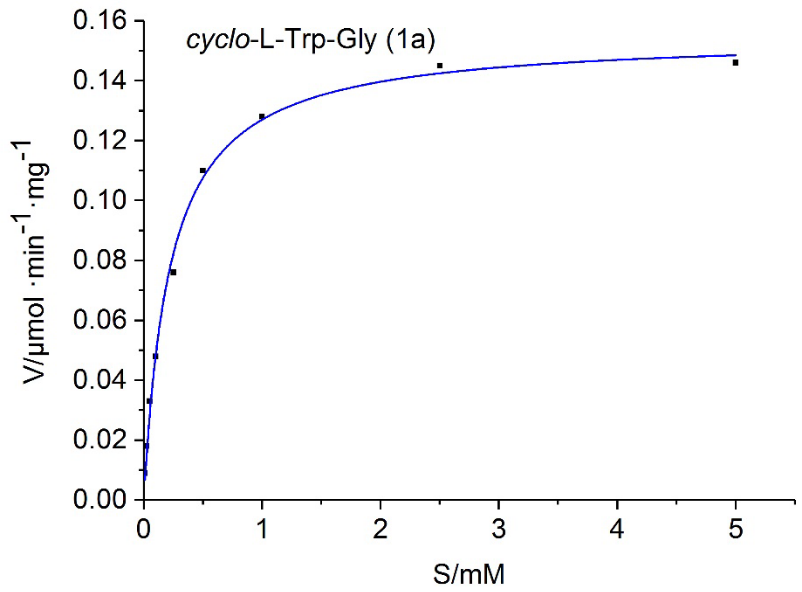

Michaelis-Menten equation

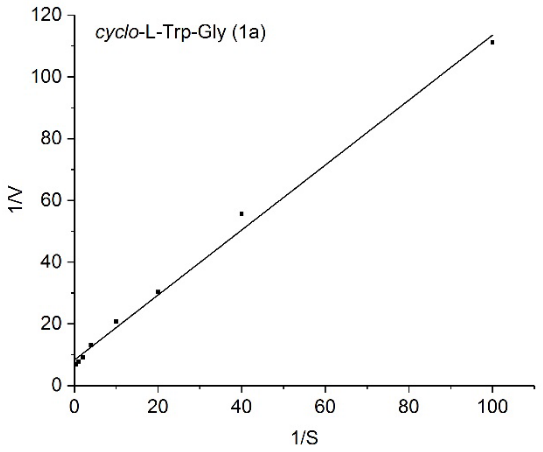

Lineweaver-Burk

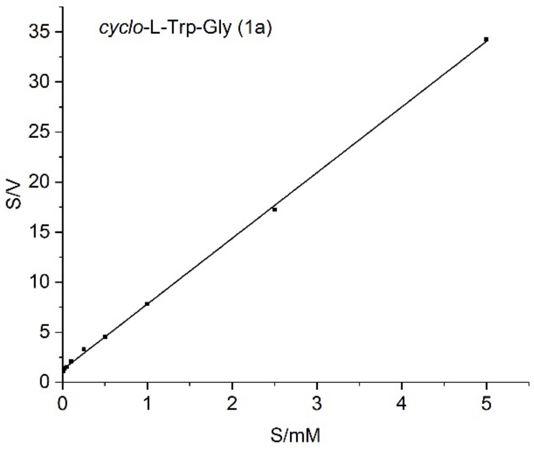

Hanes-Woolf

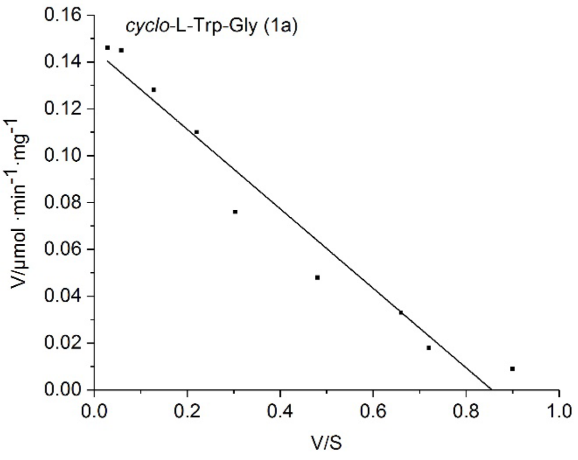

Eadie-Hofstee

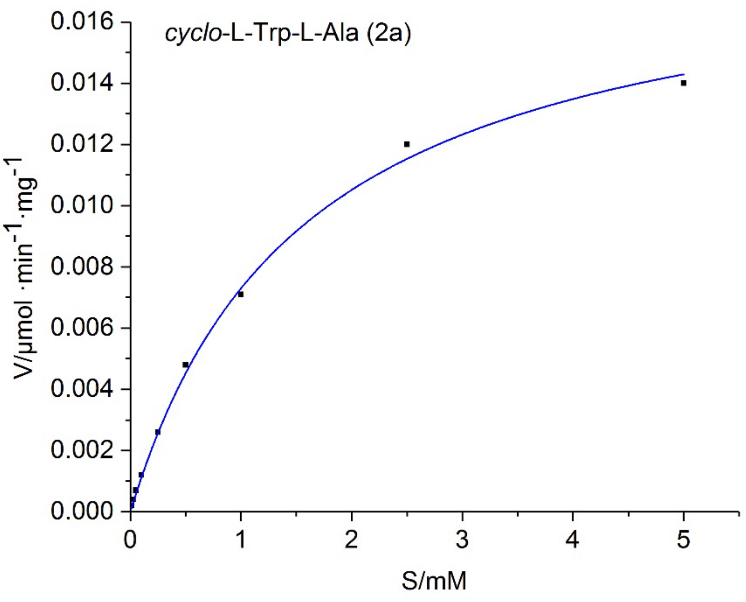

Michaelis-Menten equation

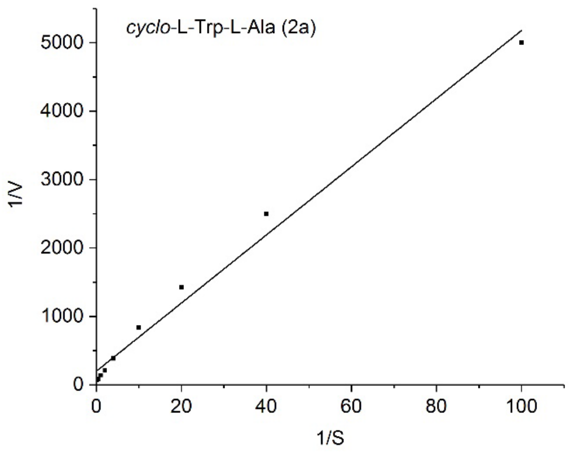

Lineweaver-Burk

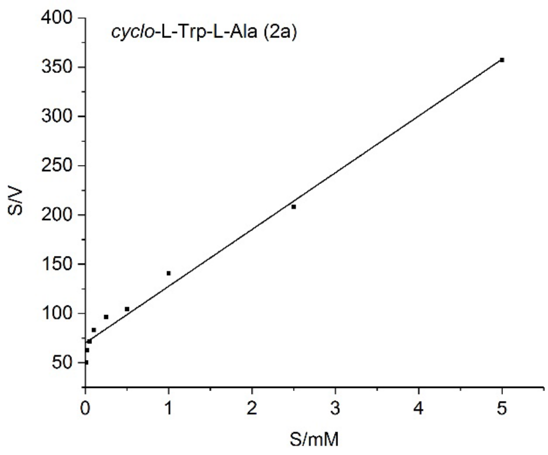

Hanes-Woolf

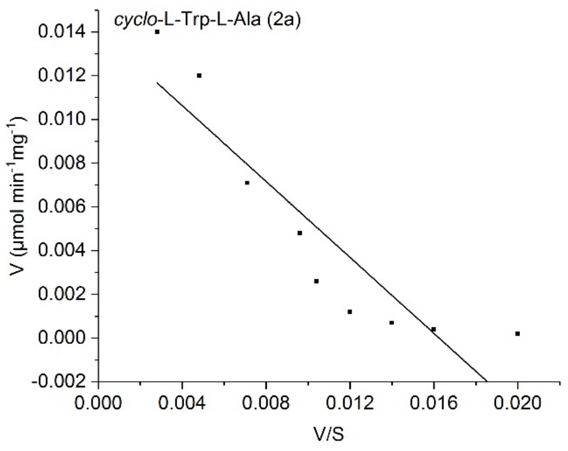

Eadie-Hofstee

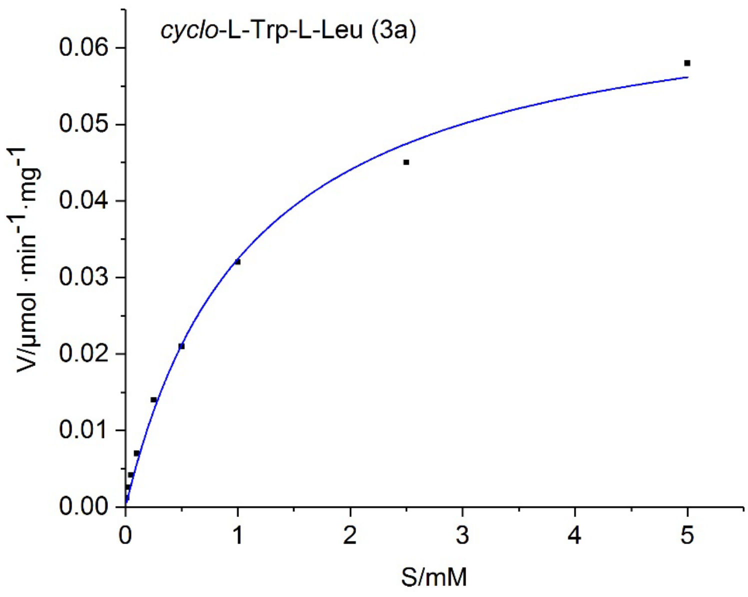

Michaelis-Menten equation

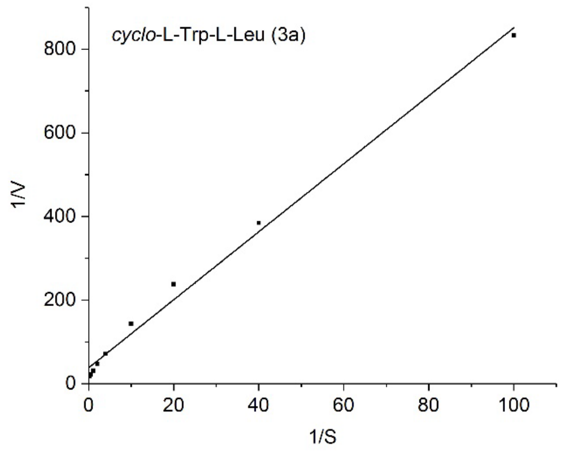

Lineweaver-Burk

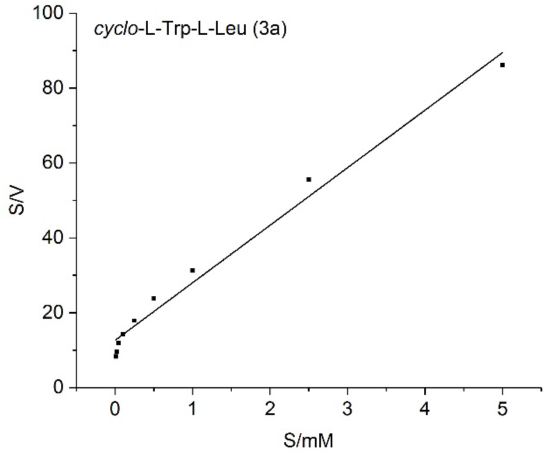

Hanes-Woolf

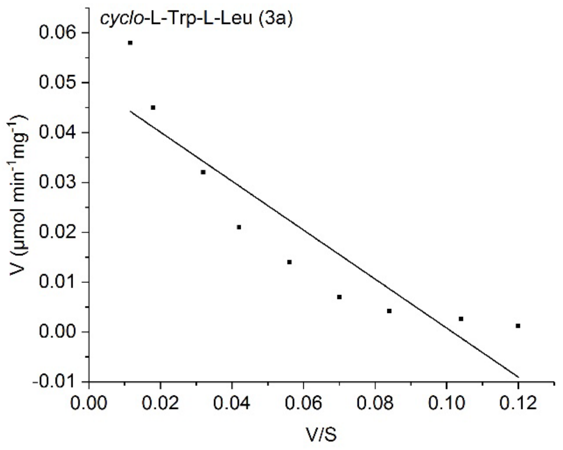

Eadie-Hofstee

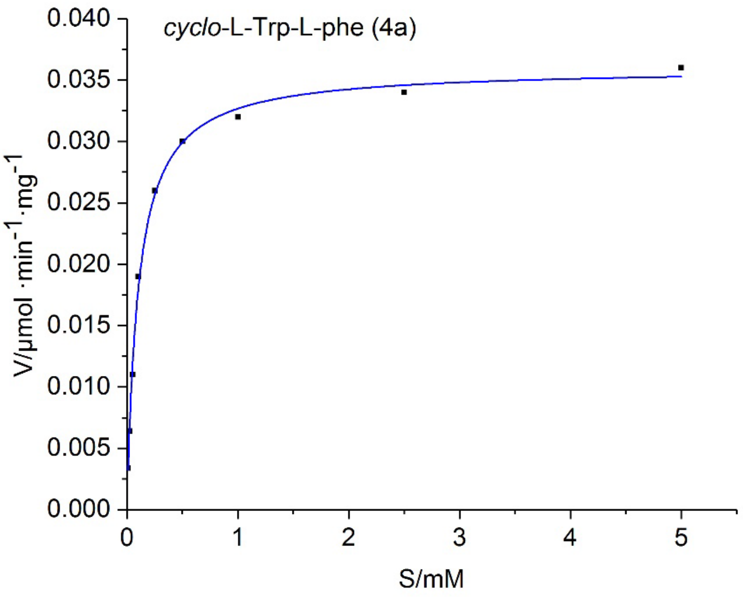

Michaelis-Menten equation

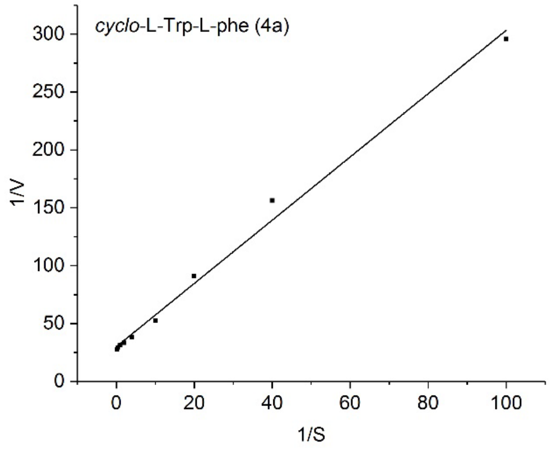

Lineweaver-Burk

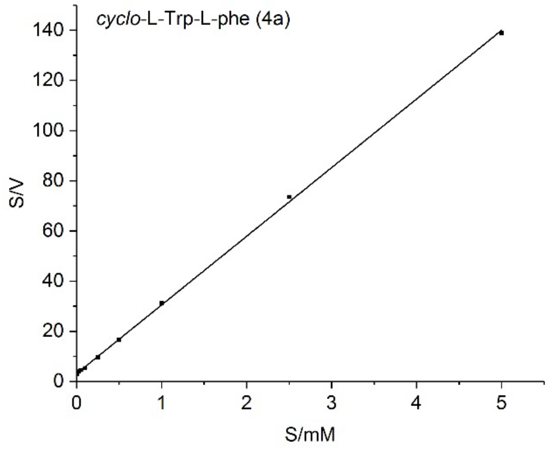

Hanes-Woolf

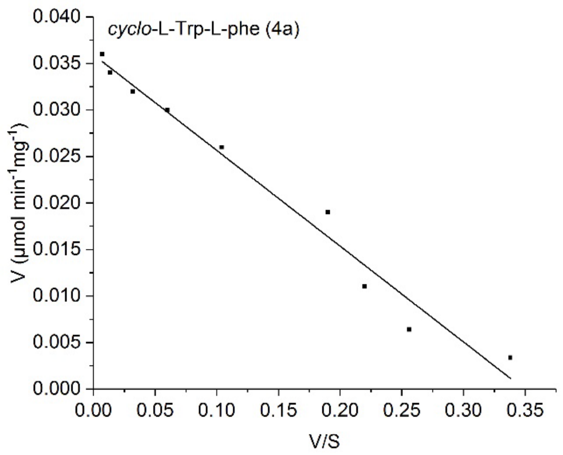

Eadie-Hofstee

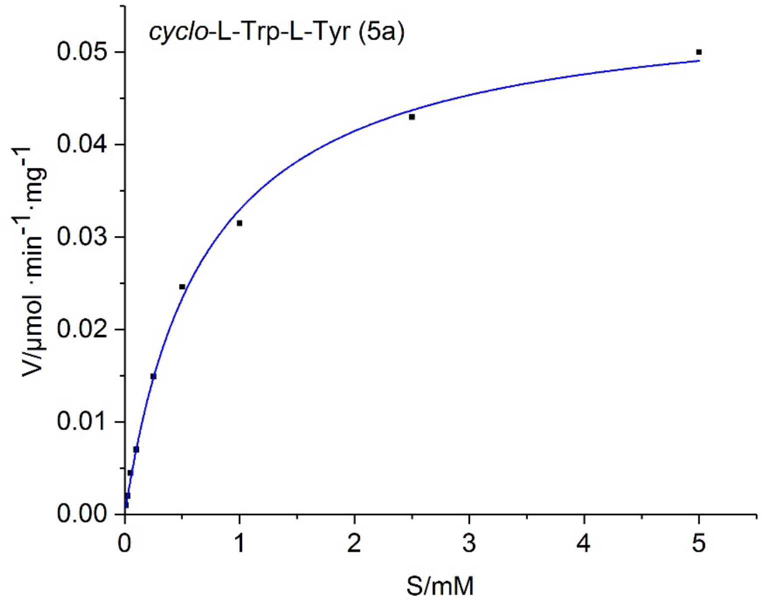

Michaelis-Menten equation

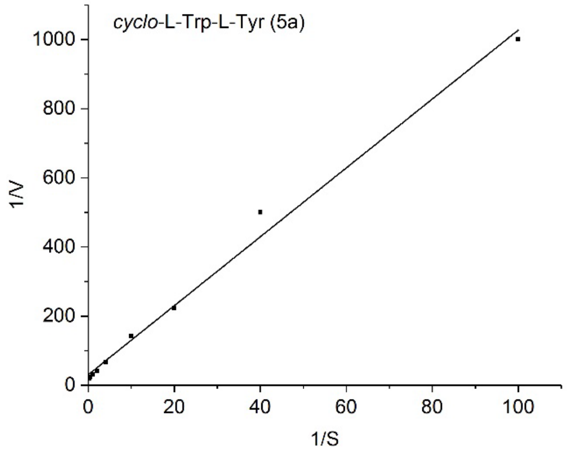

Lineweaver-Burk

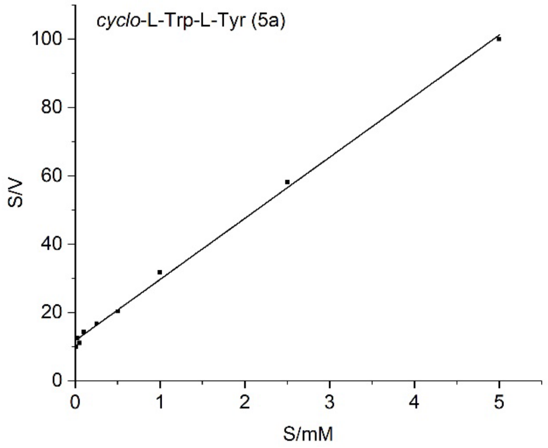

Hanes-Woolf

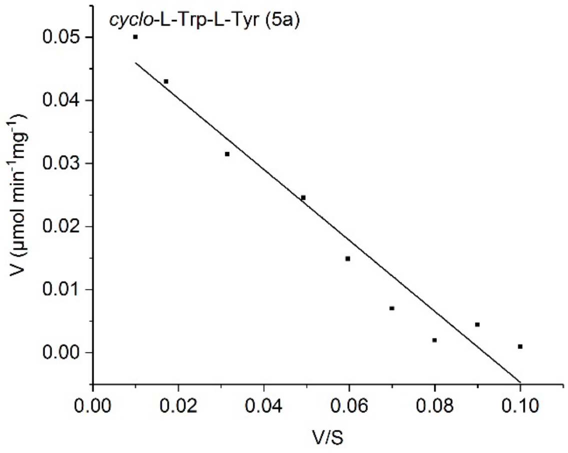

Eadie-Hofstee

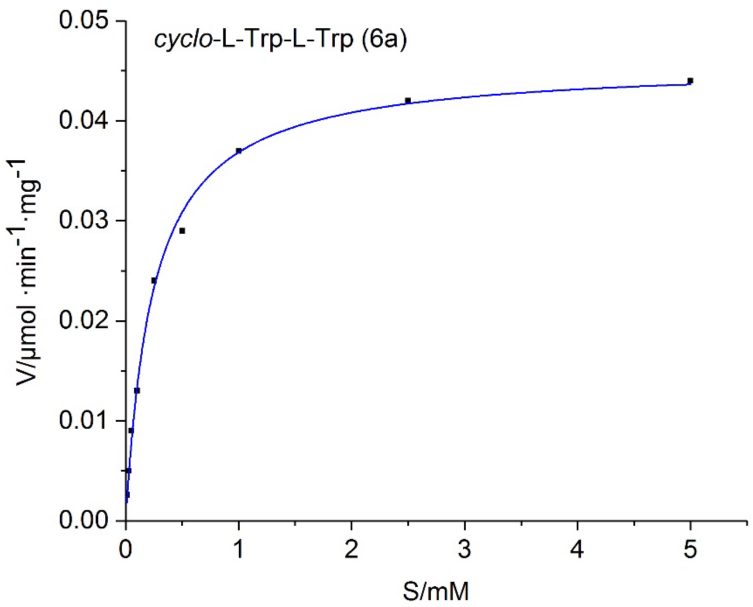

Michaelis-Menten equation

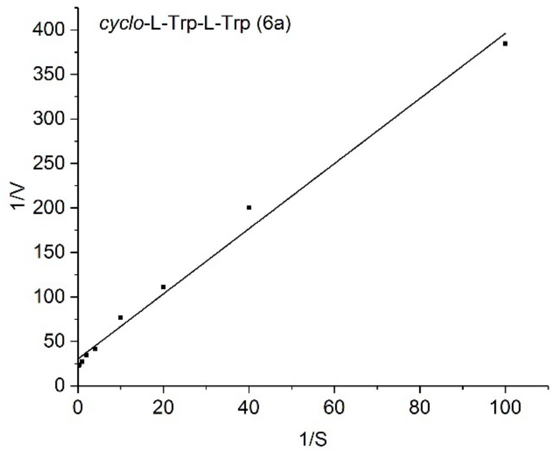

Lineweaver-Burk

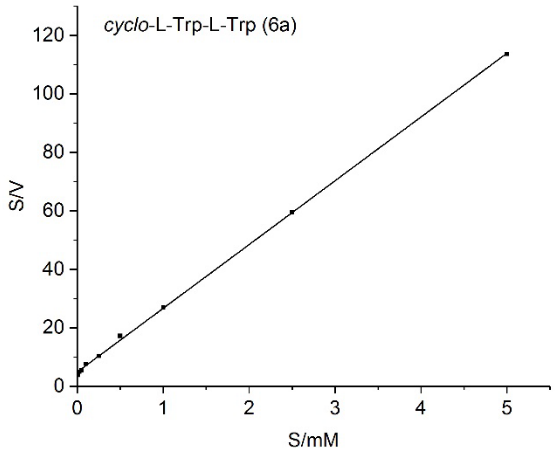

Hanes-Woolf

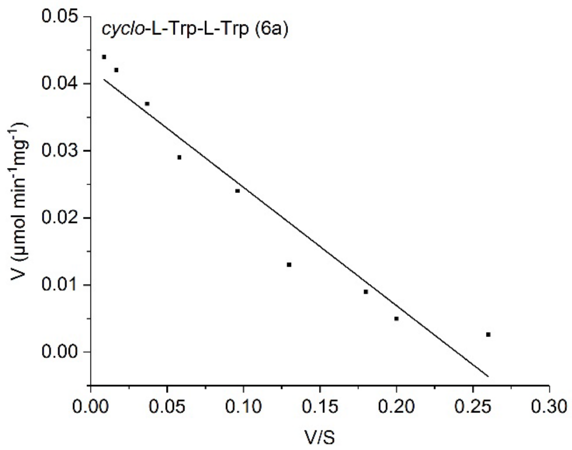

Eadie-Hofstee

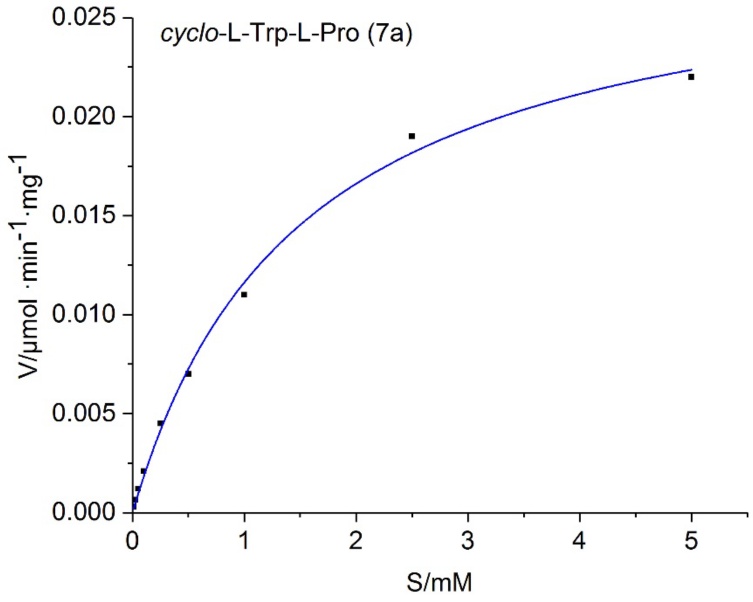

Michaelis-Menten equation

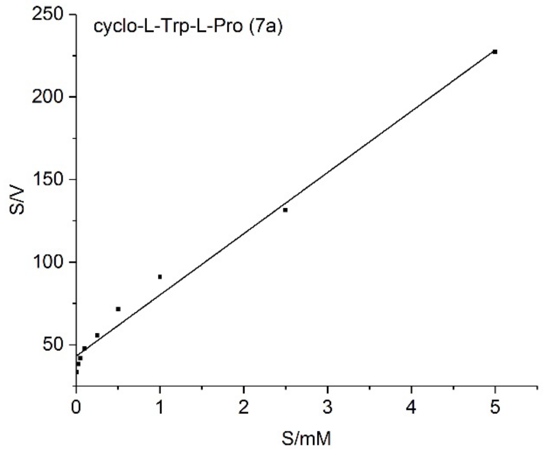

Lineweaver-Burk

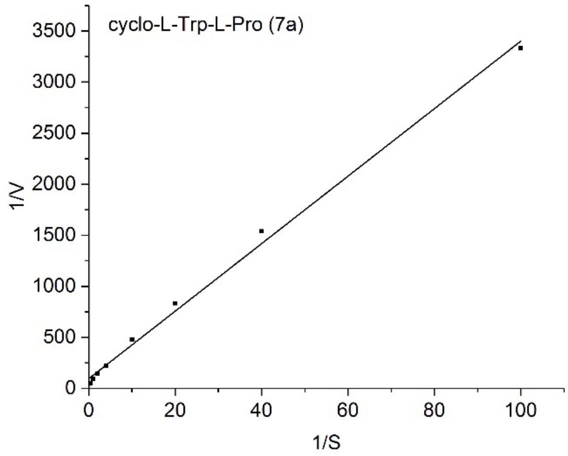

Hanes-Woolf

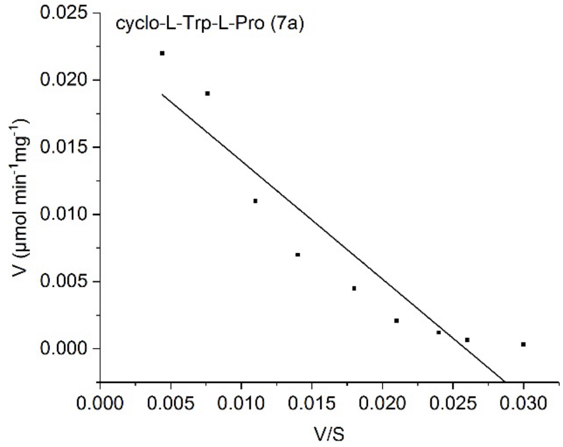

Eadie-Hofstee

Figure S2. Enzyme properties of 7-DMATS. (A) Effects of enzymatic reaction time on 7-DMATS activity; (B) pH-Dependence of 7-DMATS activity. (C) Effects of temperature on 7-DMATS activity. (D) Divalent cation requirement on 7-DMATS activity.

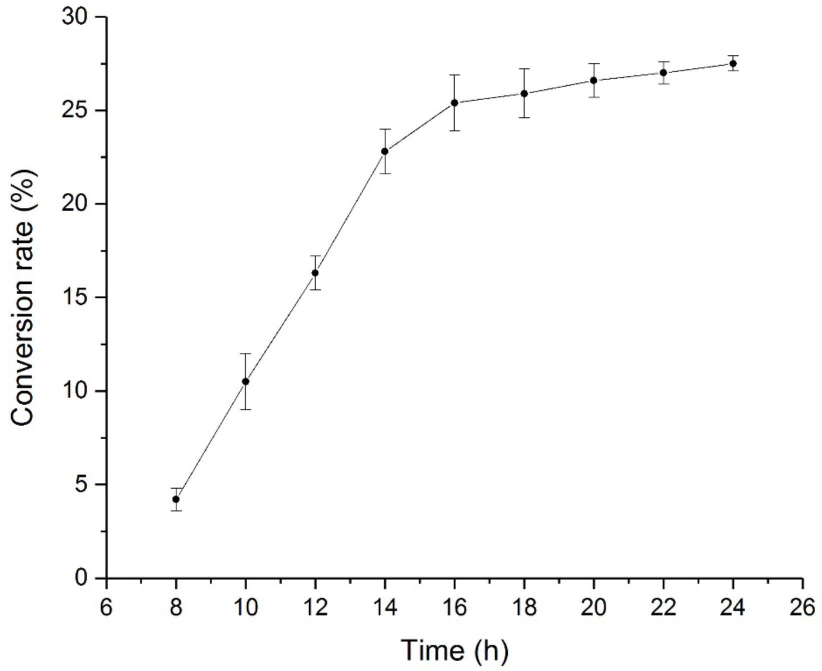

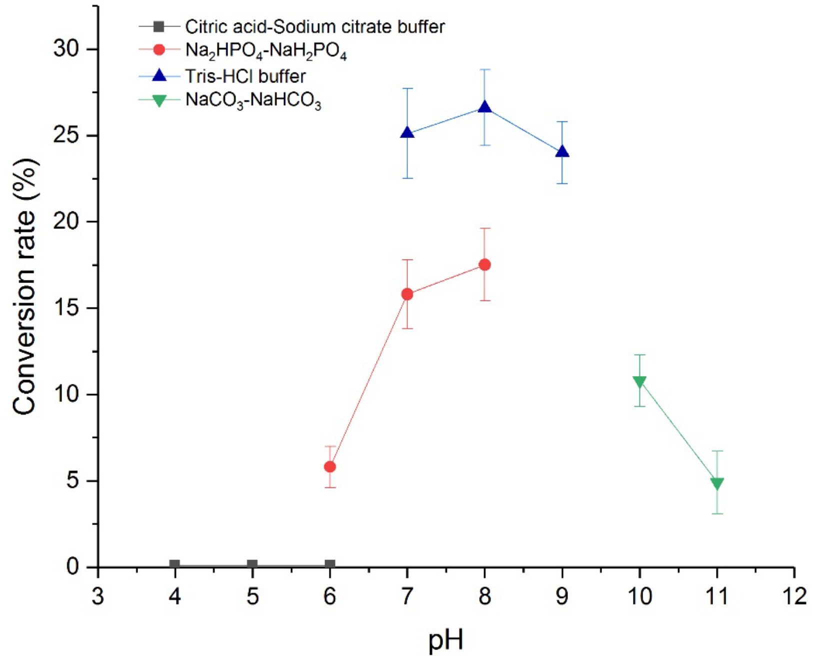

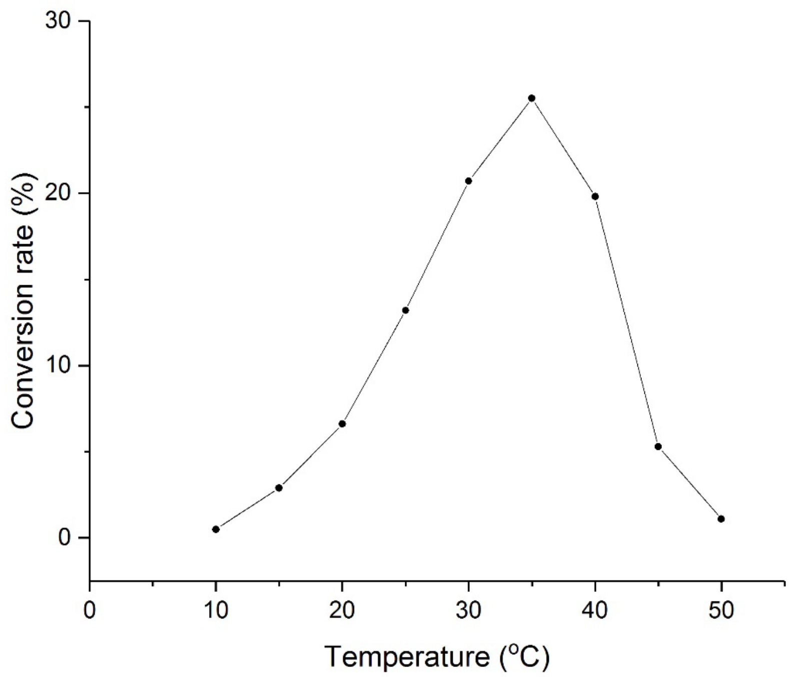

30
20
10
0
0
10
20
30
40
50
Conversion rate (%)
Temperature (°C)

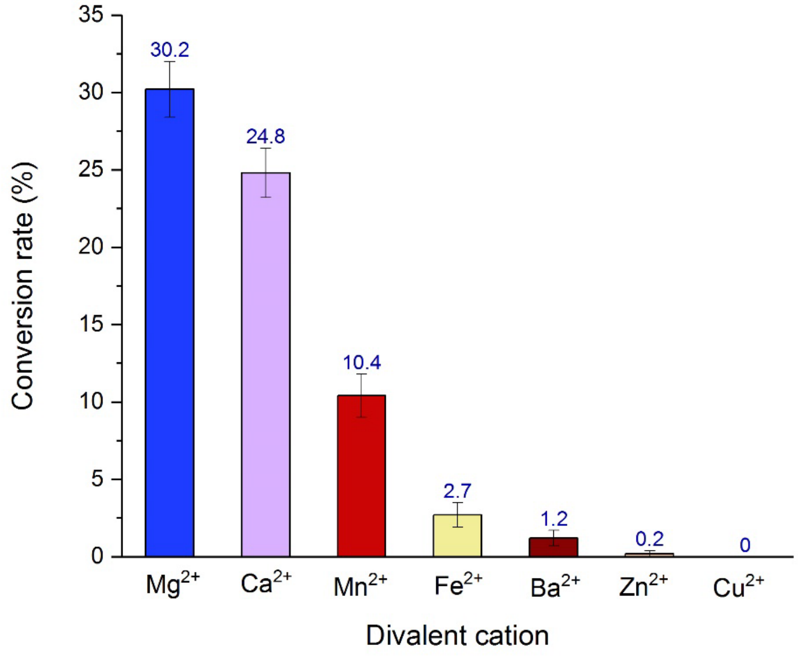

35
30
25
20
15
10
5
0
30.2
24.8
10.4
2.7
1.2
0.2
0
Conversion rate (%)
Mg2+
Ca2+
Mn2+
Fe2+
Ba2+
Zn2+
Cu2+
Divalent cation
